# Multimodal analysis of neural signals related to source memory encoding in young children

**DOI:** 10.1016/j.dcn.2025.101580

**Published:** 2025-06-13

**Authors:** Yuqing Lei, John Richards, Fengji Geng, Tracy Riggins

**Affiliations:** a Department of Psychology, University of Maryland, College Park, MD, USA; b Department of Psychology, University of South Carolina, Columbia, SC, USA; c Zhejiang University, Hangzhou, Zhejiang, China

**Keywords:** Source memory, FMRI, EEG, Multimodal, Young chlidren

## Abstract

The emergence of source memory is an important milestone during memory development. Decades of research has explored neural correlates of source memory using electroencephalography (EEG) and functional magnetic resonance imaging (fMRI). However, connections between findings from the two approaches, particularly within children, remain unclear. This study identified fMRI-informed cortical sources of two EEG signals during memory encoding, the P2 and the late slow wave (LSW), that predicted subsequent source memory performance in a sample of children aged 4 to 8 years. Both P2 and LSW were source localized to cortical areas of the medial temporal lobe (MTL), reflecting MTL’s crucial role in both early-stage information processing and late-stage integration of memory, and validating LSW’s suspected role in memory updating. The P2 effect was localized to all six tested subregions of cortical MTL in both left and right hemispheres, whereas the LSW effect was only localized to the parahippocampal cortex and entorhinal cortex. P2 was additionally localized to multiple areas in the frontoparietal network, suggesting interactions between memory encoding and other cognitive functions. These results reflect the importance and potential of considering both spatial and temporal aspects of neural activity to decode memory mechanisms, paving the way for future developmental research.

## Graphical Abstract

**Figure:**
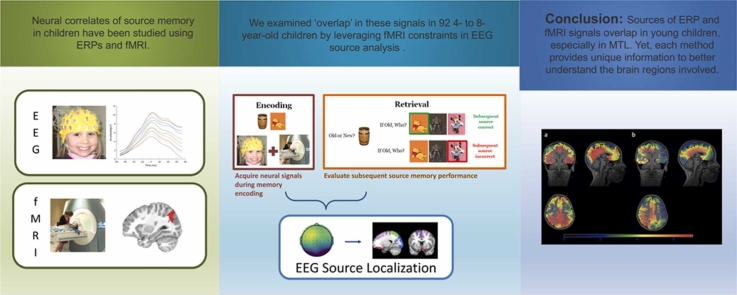

## 1 Introduction

The ability to form and recall episodic memories undergoes significant development during early childhood, due to rapid changes in the brain and associated cognitive functions during this period (Riggins, 2014). Although memory recognition capabilities emerge as early as infancy (Rose et al., 2004), episodic memory shows a more protracted developmental trajectory throughout childhood (Ghetti and Bunge, 2012), with accelerated rates of change between five to seven years of age (Riggins, 2014). Yet, the exact neural basis supporting the changes of episodic memory during this developmental window remains unclear.

Studies exploring neural correlates of episodic memory in young children often explore brain activity during the formation of memories that contain contextual details, a hallmark of episodic memory. One method to efficiently probe whether memories contain contextual information is to ask the source of the information (i.e., such as from whom or where the information was learned/acquired). Source-memory-related neural signals from EEG (Barry-Anwar et al., 2020) and fMRI measures (Ghetti and Bunge, 2012) reveal important underlying mechanisms of how the brain associates and represents context with memory items. However, signals between these different neurophysiological methods are not unified.

EEG studies using the event-related potential (ERP) approach have consistently identified a late slow wave (LSW) component in young children during memory encoding that is related to subsequent source memory performance (Riggins et al., 2013, Rollins and Riggins, 2013, Rollins and Riggins, 2018, Geng et al., 2018, Bettencourt et al., 2021). For example, Rollins and colleagues (2013) presented 6-year-old children with pictures of animals and common objects, while asking them to make a semantic judgement (i.e., animacy and size) for each picture. Source memory was assessed by asking children to subsequently recall the semantic judgements they made upon seeing those pictures again. The study found that, during memory encoding, a late-latency positive-going ERP showed significant subsequent memory effect. Consistent with this finding, Riggins and colleagues found that the amplitude of this LSW component predicted subsequent source memory recollection in children 5- to 6-year-old (Riggins et al., 2013), with LSW having a higher amplitude for subsequent source memory correct trials compared to incorrect trials.

On the other hand, fMRI studies pinpointed the medial temporal lobe (MTL) to be one of the most important brain regions for episodic memory (Ghetti et al., 2010, Ghetti and Bunge, 2012). For example, Ghetti and colleagues (2010) presented children and adolescents with drawings in either green or red during an fMRI task. Participants were subsequently asked to recall the color of each drawing. Both children and adolescents were found to engage bilateral MTL during episodic memory encoding. Additionally, MTL engagement was found to be selective for memory recollection (and not memory recognition) among young adolescents, highlighting the crucial role of MTL in forming memory details.

However, findings from these two methods remain largely separate in the field. This is unfortunate because although the measures are distinct, there are commonalities across findings suggesting that combining results could be complementary -- both measures have identified age-related changes in their respective neural signals associated with episodic memory encoding (Geng et al., 2018, Menon et al., 2005). Age-related changes in amplitude, timing, and spatial distribution of LSW are thought to underpin the growing capacity of episodic memory in young children, especially in terms of memory updating mechanisms (DeBoer et al., 2005; de Haan, 2002). However, the precise neural mechanisms generating these ERP components and their functional implications remain unclear. Differences in LSW amplitude and latency related to source memory accuracy suggest a developmental evolution in neural strategies employed during memory encoding, but the underlying cortical dynamics are not fully understood (Rollins and Riggins, 2018, Geng et al., 2018). On the other hand, from an fMRI perspective, the medial temporal lobe (MTL) shows significant age-related changes in activation during memory tasks, with increasing specialization for contextual memory encoding as children age (Menon et al., 2005, Ghetti et al., 2010). However, the nuances in the temporal dynamics of MTL engagement during memory tasks is not well characterized due to the limited temporal resolution of fMRI.

Based on the gaps identified, our study hypothesizes that the integration of ERP and fMRI data will reveal complementary insights into the neural substrates of source memory in young children. Specifically, we propose that age-related changes in MTL activation observed via fMRI are mechanistically linked to the developmental changes in the amplitude and distribution of the LSW component observed in ERP studies. We anticipate that the unique temporal information from ERPs combined with the spatial resolution of fMRI will allow us to more precisely map the neural circuitry involved in memory encoding and updating during early childhood.

This present study utilizes fMRI-informed EEG source localization to perform a multimodal neuroimaging analysis of episodic memory encoding in young children, leveraging an existing EEG and fMRI dataset collected from a sample of children aged 4 to 8 years (Canada et al., 2020, Canada et al., 2022, Geng et al., 2018, Geng et al., 2019, Geng et al., 2021, Geng et al., 2022; Riggins et al., 2018). To our knowledge, this approach has been utilized in memory research in adults (Herzmann et al., 2012; Kim et al., 2009) and infants (Reynolds and Richards, 2005, Reynolds and Richards, 2019), but not in the memory research of young children. Given that early childhood is a crucial time for memory development, uniting and discerning neural signals presented by EEG versus fMRI may be particularly important, especially since data collection is much easier for young children using the former.

It is noteworthy that memory development does not happen in a vacuum. The development of episodic memory happens along with the development of other cognitive repertoires such as attention (Cowan et al., 2024). For example, recent research has showed that attention fluctuation, coordinated by networks of regions from the frontal and parietal cortices (e.g., Marek and Dosenbach, 2018) predicts moment-to-moment memory formation especially in children (Decker et al., 2023). Thus, a widespread brain network and ERP components are implicated in episodic memory (e.g. Paller et al., 1987; DeBoer et al., 2005; Ofen et al., 2007; DeMaster and Ghetti, 2013) outside of the previously mentioned LSW and MTL. In fact, a previous analysis of the dataset used for this paper identified several cortical regions alongside the MTL that were activated during the encoding of contextual details, including the inferior/superior parietal lobule (IPL/SPL), the inferior temporal gyrus (ITG), the inferior occipital gyrus (IOG), bilateral inferior frontal gyrus (IFG), the orbital frontal gyrus (OFG) and the fusiform gyrus (FuG; Geng et al., 2019). Among these regions, IPL/SPL is considered to contribute to the visual processing of the stimuli, whereas IFG, IOG, ITG and FuG are related to the transformation from visual input to memory representations, as well as complex organization processes. In terms of EEG signals, in the re-analysis of the data for this report, we additionally identified the P2 component that was associated with subsequent episodic memory performance. Importantly, the selection of the P2 component was not based on the experimental condition differences themselves. The P2 component is a positive voltage deflection occurring approximately 150–250 ms after stimulus onset in bilateral frontal recording sites (e.g. Potts, 2004; Smith, 1993; Spitzer et al., 2008). P2 component is typically associated with allocation of attentional resources and early processing of information in infants (discussed by de Haan, 2002) and adults (e.g. Cooper et al., 2006; Freunberger et al., 2007; Potts, 2004; Smith, 1993; Wolach and Pratt, 2001), as well as contextual processing in adults (Cao et al., 2023). Thus, this report considers both MTL regions and frontal regions, as well as both LSW and P2.

In the present report, we applied source localization techniques to explore the cortical origins of two ERPs, P2 and LSW, and compared neural sources identified by the two modalities (EEG and fMRI) that were associated with successful source memory encoding in 4- to 8-year-old children. This multimodal approach benefits from information with distinct spatial and temporal resolutions, and through different neurophysiological processes, by integrating both EEG and fMRI measures. Our integration provides an opportunity to overcome the limitations of each technique and to provide a more comprehensive understanding of the neural correlates of source memory in young children.

## 2 Methods

### 2.1 Participants

Participants included typically-developing 4- to 8-year-old children. Data were derived from the first wave of a published research study examining the development of the brain in relation to memory (n = 200; Canada et al., 2020, Canada et al., 2022; Geng et al., 2018, Geng et al., 2019, Geng et al., 2021, Geng et al., 2022; Riggins et al., 2018). Prior to data collection, all methods were approved by the University’s Institutional Review Board. The majority of the children included were Caucasian (57 %), from middle- to high-income households (median income greater than $105,000 per year). A total of 95 children (40 reported females, 55 reported males, mean age 6.46 years) provided usable ERP data for source localization analysis (83 were not administered the task, 22 were excluded due to lack of usable ERP data or lack of trials in either memory condition). Ninety-two out of 95 participants simultaneously provided usable structural MRI data. An fMRI-derived mask containing six cortical regions of interest was obtained from a subset of 33 participants and was described in Geng et al., (2019).

### 2.2 Materials and procedures

Children visited the laboratory twice, approximately seven days apart. During the first visit, they completed an MRI scan including a 4-minute structural MRI sequence and a 7-minute task-based fMRI sequence during the encoding phase of the source memory task. During the second visit, they completed the same source memory encoding task with a different set of stimuli, while EEG activity was recorded. There were four exceptions where the participant performed the MRI session before their EEG session.

**2.2.1 Source Memory Task** (adapted from Ghetti et al.,2011; see Geng et al., 2018, Geng et al., 2019, Geng et al., 2022). A total of 160 images of animals and objects determined to be age appropriate were selected from the Bank of Standardized Stimuli (BOSS; Brodeur et al., 2010). All 160 possible items were randomly divided into four sets of 40 items and then counterbalanced across participants. Three of the four sets were each paired with a cartoon character (i.e., The Little Mermaid, SpongeBob, or Mickey Mouse) well known to children. The last set was used as “new items” in the retrieval phase.

Participants underwent a brief training session to ensure that they understood the task requirements. Following training, participants practiced both the encoding and retrieval portions of the task paradigm. During encoding practice, each character was paired with five different items, and participants were instructed to observe and remember which items went with which characters. During retrieval practice, participants were required to make item and source memory judgments on the 15 old items and five new items. Participants needed to obtain an accuracy score of 80 % or higher before proceeding. If they did not pass with the required accuracy, the experimenter explained the task rules again and participants were asked to complete another practice session with different stimuli. Stimuli used in training and practice trials were not used during the main study.

During encoding of the main task, participants were shown 120 pictures of objects paired with one of three characters (40 pictures per character block). Within each character block, only one character was presented. Item presentation progressed automatically with items presented for 1500 ms and an interstimulus interval ranging from 1000 to 3000 ms, with an average time of 2000 ms. The encoding phase lasted about 7 min.

The retrieval phase of the main task began approximately 15 min after the encoding phase for both fMRI and EEG. A total of 160 items (120 old and 40 new items) were presented to children during retrieval. Children were asked to identify whether they were old or new (item memory), and for old items, with which character the item had been presented (source memory). Items remained on the screen until children identified them as old or new. For old items, the three characters remained on the screen until children indicated to which character the item "belonged." All answers were given verbally, and responses were recorded by the experimenter.

#### 2.2.1 EEG recording and preprocessing

A BioSemi Active 2 EEG recording system was used to measure data continuously from 64 Ag/AgCI EEG electrodes and two vertical and two horizontal electrooculogram channels at a sampling rate of 512 Hz. Data were preprocessed using procedures described in the previous publication (Geng et al., 2018). In summary, bad channels were interpolated and ocular artifacts were corrected using the spherical spline interpolation tool and the automatic artifact correction tool provided by the Brain Electrical Source Analysis software (BESA; MEGIS Software GmbH, Gräfelfing, Germany). A maximum of 8 bad channels were allowed for inclusion in the dataset. Data was filtered, denoised, and re-referenced in EEGlab (Delorme and Makeig, 2004). EEG data were filtered with a high pass of 0.1 Hz and a low pass of 30 Hz. Data were initially segmented into epochs starting 100 ms before and ending 1500 ms after stimulus onset. Segments were rejected if any artifact was detected or if the maximum amplitude > 200 μV or if the minimum amplitude < −200 μV. Data was referenced to Pz during recording and referenced to average offline.

#### 2.2.2 MRI data acquisition and preprocessing

Participants underwent scanning in a Siemens 3.0-T scanner (MAGNETOM Trio Tim System, Siemens Medical Solutions, Erlangen, Germany) using a 32-channel coil. A T1-weighted magnetization prepared rapid gradient echo sequence was completed with a duration of 4 min and 26 s (TR 1.9 s, TE 2.32 ms, slice thickness 0.9 mm with no gap, voxel size 0.9 mm × 0.9 mm × 0.9 mm, voxel matrix 256 × 256 mm, flip angle 9°, field of volume 230 × 230 mm), during which the participants watched a movie of their choice. Task fMRI data were collected while participants completed the encoding part of the source memory task using a T2*-weighted gradient echo-planar imaging sequence, with a duration of 7 min and 6 s. Group level fMRI results have been previously analyzed and described in a previous publication from our lab (Geng et al., 2019). In summary, task fMRI data were preprocessed through slice timing correction, motion correction, normalization smoothing, coregistration and brain extraction using the Advanced Normalization Tools (ANTs; Avants et al., 2011), AFNI (Cox, 1996), Freesurfer 5.1 (Fischl, 2012), FSL (Smith et al., 2004), SPM8 (Wellcome Trust Centre for Neuroimaging, UCL, London, UK), and customized scripts.

### 2.3 Behavioral analysis

See Appendix A.

### 2.4 ERP analysis

We identified the components based on visual inspection, following published guidelines (Barry-Anwar et al., 2020, p. 31; Guy et al., 2021, Table 1). Specifically, an independent inspector reviewed the grand average ERP epochs (collapsed across all trials and conditions) to identify appropriate time windows for the P2 and LSW components, following the "collapsed localizer" approach described by Luck and Gaspelin (2017). P2 and LSW components were then separately quantified for each participant. A 50ms-long early frontal positivity was extracted, centered around the median peak amplitude during typical P2 time range (between 120 and 250 ms), for each participant across trials to correct for fluctuation and asynchrony across participants. A 50ms-long late frontal positivity was extracted and centered around highest average amplitude across participants (1200 ms) during LSW time range (1100–1500ms; Geng et al., 2018; Bettencourt et al., 2021). These short epochs were to spotlight strongest ERP activity during task trials. The resulting P2 and LSW components were re-baseline corrected to the last negative component ahead (P2: 130 −140 ms; LSW: 610–620 ms) for source localization analysis, respectively, to ensure accurate source solutions during source analysis (Pascual-Marqui et al., 2011). Repeated-measures ANOVAs were conducted on mean amplitudes for epochs associated with the P2 and LSW components using a 2 Condition (subsequent source correct, subsequent source incorrect) × 3 Coronal Plane (frontal, central, posterior) × 3 Sagittal Plane (left, middle, right) design. Condition, Coronal Plane, and Sagittal Plane were included as within-subject factors. Only main effects and interactions involving Condition are reported. Interaction effects were further examined using post hoc analysis of estimated marginal means, pairwise tested and corrected through the Tukey method (Tukey, 1991).

**Table 1:** All regions of interest for the analysis of CDR data.

*ROI*	*Brainnetome Label*	*Area*
**Brainetome-based MTL ROIs**		
rostral PhG	109,110	rostral parahippocampal gyrus (L,R)
caudal PhG	111,112	caudal parahippocampal gyrus (L,R)
lateral PPHC	113,114	lateral posterior parahippocampal gyrus (L,R)
EC	115,116	entorhinal cortex (L,R)
TI	117,118	temporal agranular insular cortex (L,R)
medial PPHC	119,120	medial posterior parahippocampal gyrus (L,R)
**fMRI-constrained ROIs**		
left IPL/SPL	/	left inferior/superior parietal lobule
IOG	/	inferior occipital gyrus
left ITG	/	inferior temporal gyrus
left IFG	/	inferior frontal gyrus
FuG	/	fusiform gyrus
OFG	/	orbital frontal gyrus

### 2.5 EEG source localization analysis

#### 2.5.1 Head model construction

Individual structural T1-weighted images were used to generate head models for each participant. Age-specific MRI templates were applied during brain extraction (Richards et al., 2016) to maximum volumes of extracted cortical tissues. Brain segmentation was completed using FSL (Smith et al., 2004) and Freesurfer (Dale et al., 1999). Each head model consisted of nine segmented head compartments of different types of media with corresponding estimated electric conductivities to create realistic head models: grey matter, white matter, cerebrospinal fluid, skull, muscle, dura, eyeballs, nasal cavity, and scalp (see Reynolds and Richards, 2009). Anatomical regions of eyeballs and nasal cavities (i.e., fiducials) were manually identified for each participant in MRIcron (Rorden and Brett, 2000). Grey matter, white matter, and CSF were extracted using FSL’s Brain Extraction Tool (BET). Scalp, skull, muscle and dura volumes were created using ANTs’ ImageMath function, FSL’s fslmaths function, and customized scripts.

#### 2.5.2 Fiducial landmark marking

Three-dimensional coordinates of nine anatomical fiducial landmarks were manually marked on each participant’s structural MRI scans using MRIcron (Rorden and Brett, 2000), including the anterior commissure, the posterior commissure, the nasion, the inion, the left mastoid, the right mastoid, the left pre-auricular, the right pre-auricular, and the vertex. The first two anatomical landmarks are used to set up MRI coordination system, while the rest are commonly used for the 10–10 system electrode placement (Richards et al., 2015). The physical location of these anatomical landmarks on individual structural MRI scans were used to spatially re-align EEG electrode placement and MRI data within each participant. These procedures were to improve result accuracy and to account for individual variability in electrode placement as well as head shapes.

#### 2.5.3 Source reconstruction

The source localization algorithm (adapted from Conte and Richards, 2022) was applied to individual head spaces from brain-extracted structural images. Source reconstruction procedures was performed using customized scripted adapted from Fieldtrip functions (Oostenveld et al., 2011). The source model was constructed as 3 mm grids of grey matter and eyes within each participant’s MRI, representing possible electric sources of observed ERPs (measured by current density). The segmented head model was constructed as a tetrahedral mesh of the head using the finite element method (FEM; Awada et al., 1997; for a discussion of geometry choices during mesh construction see Conte and Richards, 2022). Each tetrahedron was assigned to one of the nine head media described above. The lead field matrix (i.e., the forward model) integrated the source model, the head model, and re-aligned EEG electrode locations to simulate the process of electrical current within cortices flowing through layers of the head, projecting electrical activities onto the scalp that were subsequently captured by EEG electrodes (Hallez et al., 2007). The inverse model utilized the eLORETA method (Pascual-Marqui et al., 2011), producing a dipole moment vector at each 3 mm source grid. A current density reconstructed (CDR) score was estimated for each source grid from the magnitudes of the dipole moment vectors, separately for P2 and LSW activity and for each condition. CDR scores measure the magnitudes of the 3-dimention vector solutions at each source grid and represent average source strength during entire epoch of ERP. eLORETA method is designed to minimize total estimated squared current density, thereby offering a solution for the inverse model without requiring a priori information about the number of sources, allowing us to compute one average CDR score for each anatomical source ROI.

Anatomical source ROIs were constructed in individual head spaces using the Brainnetome atlas (Fan et al., 2016) and published fMRI results from this dataset (Geng et al., 2019). The Brainnetome Atlas offers greater detail (210 regions of cortical parcellation) of the brain compared to other commonly used brain atlases (e.g., the Desikan-Killiany atlas has 84 regions, the Yeo 2011 atlas has 17 regions). In addition to finer parcellation, it also bases the parcellation on both anatomical features and connectional architecture of the brain, allowing for more accurate parcellation across diverse brain shapes. Though the atlas is not created from children’s brain images, brain atlases created from adult brains are commonly used in developmental research, due to the lack of age-specific atlases (Altman and Bernal, 2011). Note that there are a few age-specific pediatric atlases out there, such as the UNC Human Brain Atlas (UNC Neuro Image and Analysis Laboratories, 2009), but they are not appropriate ages for this study.

Average CDR scores for each ROI source area were compared between subsequent source memory correct versus source memory incorrect trials using paired t-tests. Age-related effects were examined using mixed-effect linear models; findings are reported in Appendix B. Multiple comparison correction was conducted using BH method to adjust for FDR. Left and right hemispheres were tested and corrected separately if the ROI is bilateral.

We focused on the two sets of source ROIs described above: 1) cortical MTL derived from the Brainnetome atlases and 2) fMRI-constrained ROIs (details below). All ROIs are listed in Table 1.

#### 2.5.4 MTL ROIs

CDR scores within the cortical areas of all Brainnetome MTL subregions were tested bilaterally. Tested areas include various parts of the parahippocampal gyrus (PhG), the entorhinal cortex (EC), and the temporal agranular insula (TI).

#### 2.5.5 fMRI-constrained ROIs

A task-specific group mask was achieved from the whole-brain analysis of 33 participants’ fMRI data and was described in a previous paper (Geng et al., 2019). Note that EEG and fMRI mask are not obtained from the exact same group of children but are from the same project within the same age range. In short, BOLD signal was compared between subsequent source correct and subsequent source incorrect trials. Six cortical regions were identified through whole-brain analysis that showed subsequent source memory effect. These regions are left-hemisphere IPL/SPL, IOG, left-hemisphere ITG, left-hemisphere IFG, FuG and OFG.

These a priori identified cortical regions were transformed to subject head spaces to inform our source model construction for ERP source localization. The MNI-space group mask was upsampled from 3 mm to 1 mm resolution and realigned to brain-extracted individual structural MRI scans using AFNI’s 3dAllineate function. This transformation yielded one fMRI ROI mask for each participant, in their individual head space. All individual ROI masks were visually inspected.

## 3 Results

### 3.1 ERP results

#### 3.1.1 P2

Analysis of the P2 component showed a significant interaction between Condition and Sagittal plane, *F*(2, 188) = 5.93, *p* = 0.003, as well as a three-way interaction between Condition X Coronal plane X Sagittal plane, *F*(4, 376) = 3.87, *p* = 0.043. To further explore these interactions, post hoc pairwise testing was conducted in each Coronal plane X Sagittal plane clusters. In frontal (*t*(396) = 2.99, *p* = 0.003) and central (*t*(396) = 2.39, *p* = 0.017) clusters, P2 showed a significant subsequent source memory effect (greater amplitude to subsequent source correct versus source incorrect trials) in left, but not middle and right channels. In the central cluster, P2 amplitude showed the opposite effect (i.e. significant decrease in source correct condition, compared to source incorrect condition) in middle (*t*(396) = -2.88, *p* = 0.004) and right (*t*(396) = -2.15, *p* = 0.03) channel clusters. In the posterior area, P2 amplitude also showed the opposite source memory effect in right channel clusters, *t*(396) = -2.28, *p* = 0.023).

#### 3.1.2 LSW

As illustrated in Fig. 4c, Analysis of the LSW component also indicated a significant interaction between Condition and Sagittal plane, *F*(2, 188) = 4.02, *p* = 0.020. Post hoc pairwise testing of this interaction showed that left (but not middle and right) LSW was significantly greater for subsequent correct source memory, compared incorrect source memory, *t*(217) = 2.30, *p* = 0.023.

**Fig. 1:**
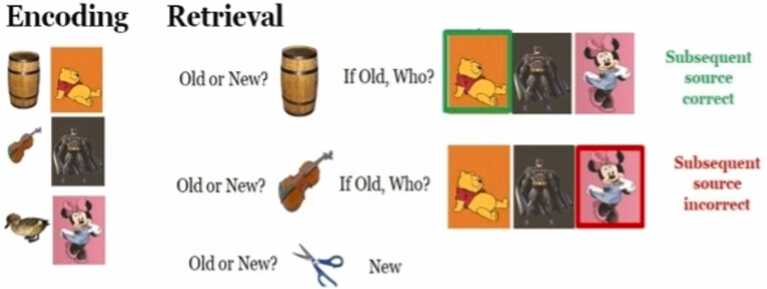
Task paradigm of the source memory task (Geng et al., 2018, Geng et al., 2019, Geng et al., 2022). during the encoding phase, each item is associated with one of three cartoon characters that are familiar to young children (Winnie the Pooh, Batman, and Minnie Mouse in version 1, Little Mermaid, Mickey Mouse, SpongeBob in version 2). fMRI or ERP data were collected during encoding. Approximately 15 min after the encoding phase of the task, participants were asked to perform the retrieval phase outside the fMRI/ERP recording environments. In each trial, participants were first asked to identify if they had seen the presented stimulus during the encoding phase, and if they answered “yes”, they were asked to identify to which of the characters did the stimulus belong.

**Fig. 2:**
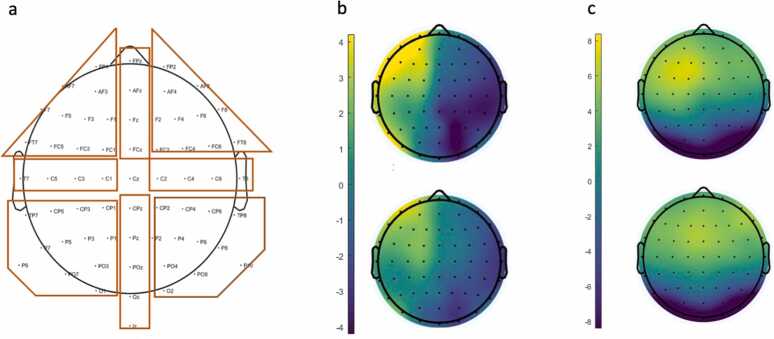
ERP signals between memory conditions. A) EEG channel locations and coronal (frontal, central, posterior) x sagittal (left, middle, right) channel clustering. B) average P2 amplitude in subsequent source memory correct (top) and incorrect trials (bottom), averaged across 150 ms around P2 peak amplitude. C) average LSW amplitude in subsequent source memory correct (top) and incorrect trials (bottom), averaged across from 1125 to 1275ms after trial onset.

**Fig. 3:**
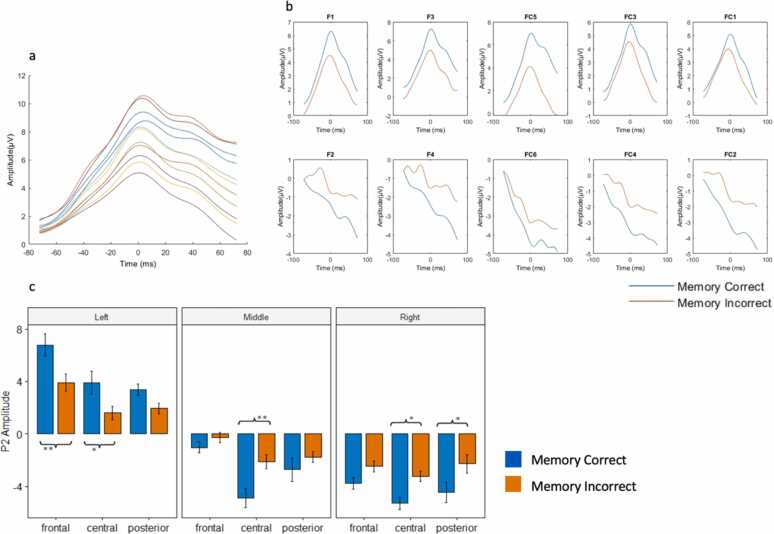
P2 component. A) P2 activity during source correct trials across all left frontal channels, centered around individual P2 peak. B) conditional differences in frontal P2 by channel, contrasting left hemisphere (top) vs. Right hemisphere (bottom) channels. C) P2 activity by coronal x sagittal clustering.

**Fig. 4:**
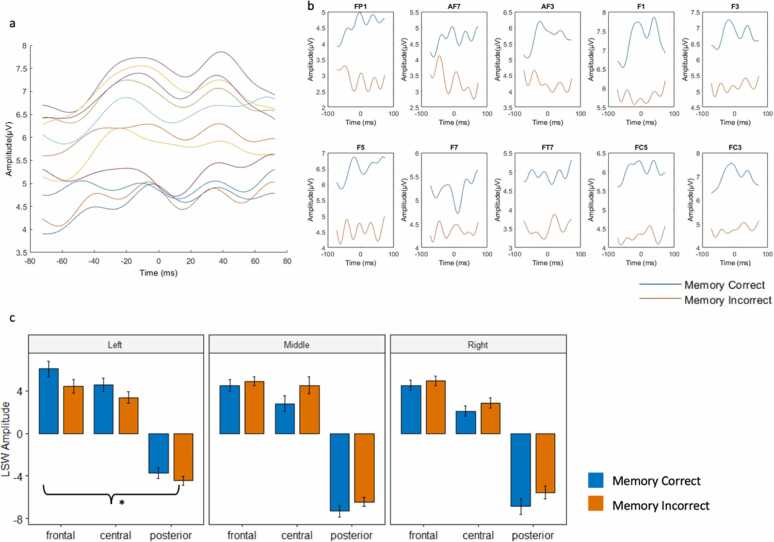
LSW component. A) LSW activity during source correct trials across all left frontal channels, centered around 1200 ms after trial onset. B) left frontal LSW activity by channel and condition. C) LSW activity by coronal x sagittal clustering.

### 3.2 Source localization results

**Fig. 5:**
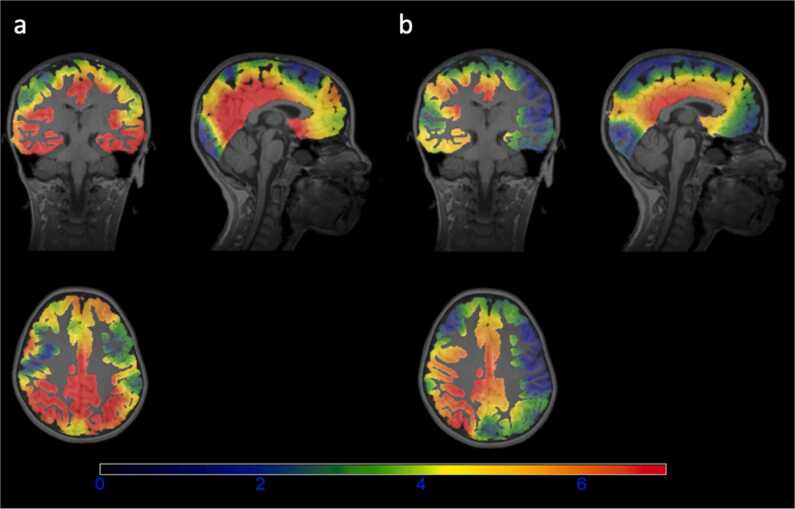
An illustration of the source localization outcome in individual head space. Panels show the average CDR scores during LSW at each source grid of the participant's head model, for a) the source memory correct and b) source memory incorrect conditions, respectively. Regions exhibiting strong conditional differences in their CDR scores (hot colors) include several frontal and parietal regions as well as MTL and its surroundings, consistent with quantitative results discussed below.

We estimated a CDR score for each source grid in individual participants’ head models based on epoched P2 and LSW activity, for source correct and source incorrect memory conditions separately. These CDR scores were then averaged across all source grids in each hypothesized ROI and compared between the two conditions (Fig. 5).

#### 3.2.1 MTL

**Fig. 6:**
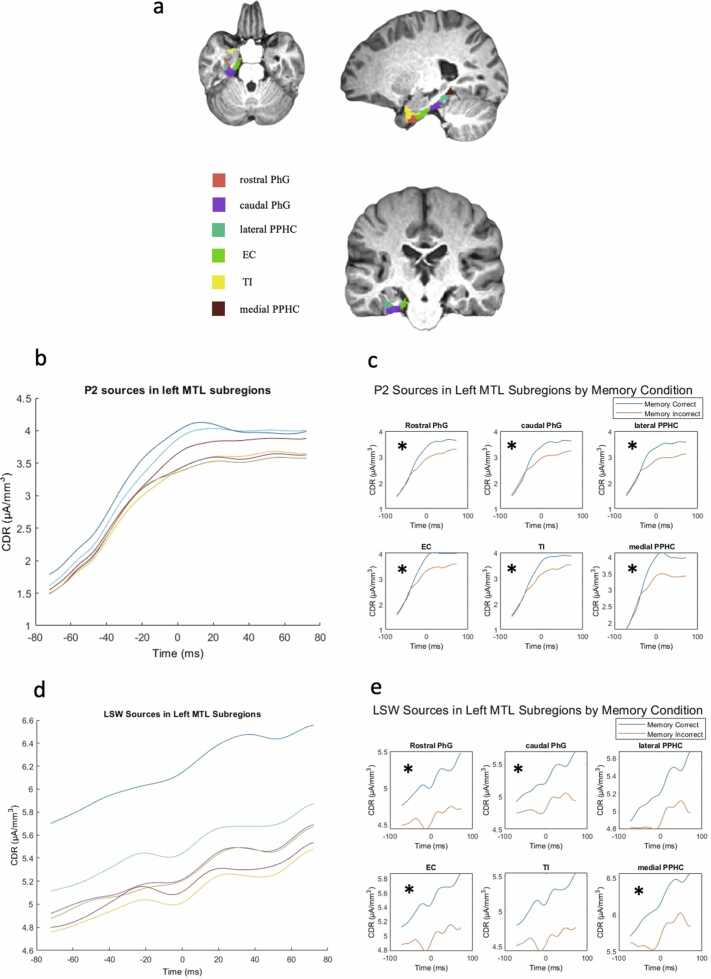
A) Subregions in left MTL. B) P2 sources across all left MTL subregions. Cortical CDR scores steadily increased until hitting peak P2 activity. C) P2 source activity by memory conditions in each left-hemisphere subregion of MTL. D) LSW sources across all left MTL subregions. Cortical CDR scores steadily increased overtime. E) LSW source activity by memory conditions in each left-hemisphere subregion of MTL. (*p < 0.05).

As illustrated in Fig. 6c, within the MTL region, multiple subregions showed significant CDR differences during peak P2 activity between source correct and source incorrect conditions. All left hemisphere subregions showed greater CDR scores in source correct condition than incorrect condition, and survived FDR corrections (left and right hemisphere were corrected separately). The areas include rostral PhG (left: *t*(91) = 0.48, *p* = 0.031; right: *t*(91) = 0.27, *p* = 0.060), caudal PhG (left: *t*(91) = 0.49, *p* = 0.034; right: *t*(91) = 0.29, *p* = 0.060), lateral PPHC (left: *t*(91) = 0.53, *p* = 0.036; right: *t*(91) = 0.30, *p* = 0.060), medial PPHC (left: *t*(91) = 0.57, *p* = 0.031; right: *t*(91) = 0.34, *p* = 0.060), EC (left: *t*(91) = 0.50, *p* = 0.031; right: *t*(91) = 0.29, *p* = 0.060), and TI (left: *t*(91) = 0.60, *p* = 0.034; right: *t*(91) = 0.46, *p* = 0.060). Right hemisphere subregions showed marginal results. No areas showed greater activity in subsequent source memory incorrect condition compared to source memory correct condition. These results indicate stronger P2-related cortical activity during memory encoding in subsequent successful retrieval of source memory.

As illustrated in Fig. 6e, LSW sources also showed conditional differences within multiple areas within MTL, including left, but not right, rostral PhG, *t*(91) = 0.54, *p* = 0.039; caudal PhG, *t*(91) = 0.44, p = 0.053; medial PPHC, *t*(91) = 0.58, *p* = 0.039; and EC, *t*(91) = 0.60, *p* = 0.039. No areas showed greater activity in subsequent source memory incorrect condition compared to source memory correct condition.

#### 3.2.2 fMRI-constrained ROIs

**Fig. 7:**
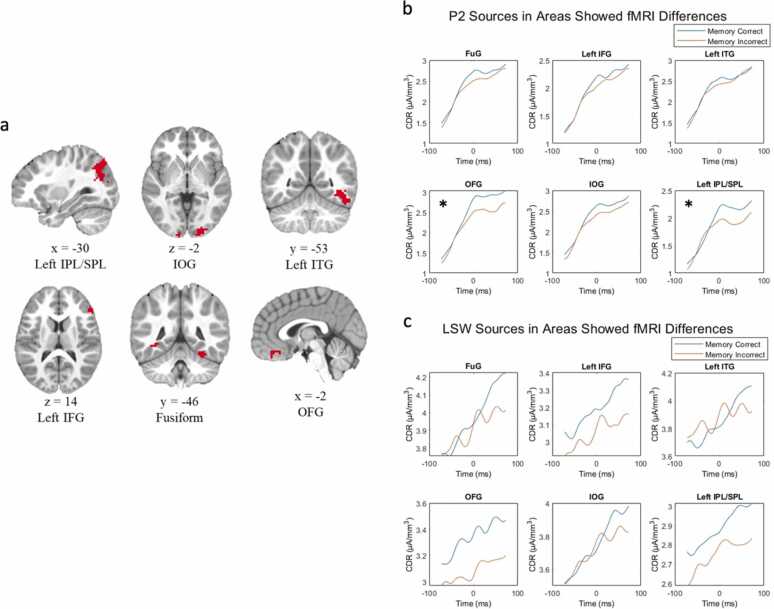
FMRI-constrained source localization. A) adapted from Geng et al. (2019). cortical regions showing greater fMRI activation in source memory correct versus incorrect condition. B) P2 source activity in each fMRI-identified ROI by source memory condition. C) LSW source activity in each fMRI-identified ROI by source memory condition.

As illustrated in Fig. 7b, within task ROIs from fMRI data, two regions showed significant CDR differences during peak P2 activity between source correct and source incorrect conditions. These two ROIs are OFG, *t*(91) = 0.36, *p* = 0.024, and left IPL/SPL, *t*(91) = 0.27, *p* = 0.024. As illustrated in Fig. 7c, LSW-related CDR scores were also significantly greater in OFG in source correct condition compared to source incorrect condition, but the result did not survive FDR correction. No areas showed greater activity in subsequent source memory incorrect condition compared to source memory correct condition.

## 4 Discussion

The goal of the current study was to examine multimodal neural signals related to episodic memory encoding in young children. We identified a LSW component, as well as a novel P2 component, that predicted subsequent source memory performance. While the P2 component is not traditionally associated with memory performance, its involvement highlights the importance of other cognitive functions that support episodic memory encoding as well as information processing. To better understand the neural origins and exact functionalities of both components, we utilized EEG source localization and projected scalp ERP potentials into individual cortical spaces and examined the underlying anatomical sources. Multiple subregions of MTL were identified as electrophysical sources of both P2 and LSW components, suggesting the crucial role of MTL in both early and late-stage memory encoding processes. fMRI-informed source localization additionally identified several cortical regions that were sources of P2, including OFG and left IPL/SPL, suggesting the engagement of frontoparietal areas in the early processing of source memory stimuli. In summary, the results leveraged both ERP and fMRI data to highlight the dynamic and multifaceted nature of episodic memory encoding in developing brain.

### 4.1 ERPs at encoding predicted subsequent source memory performance

#### 4.1.1 P2

During encoding, stimulus-induced P2 was predictive of subsequent source memory performance. The P2 component showed subsequent source memory effects in frontal and left channel clusters, and in individual frontal and frontocentral channels. Although the role of the P2 component in memory has not been previously established, existing research in adults suggests a role in early memory processing during the integration of information. For example, P2 amplitudes have been shown to vary with repetition of memory stimuli and are sensitive to item familiarity (Rugg and Curran, 2007). Specifically, P2 shows increased amplitude in response to the first memorized item during a memory encoding task compared to the rest of the items (Wolach and Pratt, 2001), and decreased amplitude to immediately repeated memory stimuli (Smith, 1993), suggesting that P2 amplitude reflects the “amount” of early processing to memory items.

Given the unique role of P2 in attention and contextual information processing, it may be crucial for encoding new information into memory. Results of the present study showed greater P2 amplitudes in left frontal regions for trials in which source information was subsequently correct, potentially reflecting the engagement of a lateralized frontoparietal network associated with attentional processes, crucial for the encoding of source information (Geng et al., 2022).

#### 4.1.2 LSW

During encoding, LSW was predictive of subsequent source memory performance. The LSW component showed greater amplitude in left versus right channel clusters. This finding is consistent with the previous analysis examining LSW in this dataset (Geng et al., 2018). However, LSW component did not show differences at individual channels after corrections for multiple comparisons. This lack of significance is likely attributed to its broad topographical distribution (Riggins et al., 2009, Riggins et al., 2013), as well as variability across individuals at different ages (Geng et al., 2018, Rollins and Riggins, 2013). Previous studies suggested that LSW may reflect the updating of memory for a partially encoded stimulus (Reynolds and Richards, 2005, Riggins et al., 2013), and thus there could also be variability in this component due to its association with later stages of memory processing, which might be more susceptible to individual differences in memory strategies.

### 4.2 Cortical sources of P2 and LSW

#### 4.2.1 MTL

Source analysis revealed that P2- and LSW- associated cortical activity showed significant effects in multiple areas of MTL. Specifically, the P2 effect was found in all six tested subregions of cortical MTL in both left and right hemispheres, while the LSW effect was only present in PhG and EC. The MTL is a key region for information integration, memory association, and updating. Importantly, the PhG is known for its role in processing environmental contexts, and the EC is the principal site of cortical input into the hippocampus (Suzuki and Amaral, 1994), heavily involved in relaying contextual information to hippocampus. Given MTL’s unique role in information integration, the P2 result adds new evidence to previous research suggesting P2’s role in the processing and integration of contextual information (Cao et al., 2023), and underscores the finding that children start to process contextual details associated with a memory item as early as 200–250ms. Additionally, the synchronous activation in MTL and the frontoparietal network during P2 suggests MTL’s role in the interplay between memory processes and frontoparietal control processes, calling for further investigation (see discussion below in Section 4.4).

The LSW result in PhG and EC supports previous work suggesting the LSW reflects activity during memory association and information updating (Nelson, 1994, Reynolds and Richards, 2005, Riggins et al., 2013), especially given the late latency it has during memory encoding. Given previously reported age-related changes in the timing and amplitude of LSW (Geng et al., 2018, Rollins and Riggins, 2013), its localized neural sources in the MTL region converge with neuroimaging findings showing decreased activation in the MTL during memory encoding as children age (Ghetti et al., 2010, Menon et al., 2005, Ofen et al., 2007). Both P2 and LSW results show that scalp EEG signals capture activities in the cortical MTL area regardless of some of its deep anatomical locations in the brain (e.g., PhG) and corroborate the important role of MTL in both early and late stages of encoding process.

#### 4.2.2 fMRI-constrained ROIs

fMRI-constrained multimodal analysis provides a more nuanced understanding of the neural underpinnings of memory encoding, demonstrating a significant overlap between localized ERP sources and fMRI ROIs. Specifically, the source memory effect during P2 was localized to two out of six fMRI ROIs that showed conditional differences in BOLD activation (i.e., the OFG and the left IPL/SPL ROIs). Both regions are considered a part of the frontoparietal network, an integral network to complex cognitive processes (Marek and Dosenbach, 2018) that is previously found to be recruited during memory formation (Geng et al., 2022). For example, existing research suggests that activities within the posterior medial cortex (i.e., precuneus/medial parietal cortex involving IPL and SPL) are found to modulate memory performance and emerges with age (Amlien et al., 2018). The LSW presented similar trends in both of these areas (Fig. 7c), however, the results did not survive multiple comparison corrections. These multimodal results suggest that although EEG and fMRI tap different neurophysiological measures, they do point to the same or shared source memory encoding mechanism. The differences in the localized sources between early stage P2 and the late stage LSW provide additional temporal information to the potentially roles of fMRI-identified areas. The P2 component, occurring early in the processing timeline, further suggests the frontoparietal network is playing a role in the early integration of sensory information, attention, cognitive strategies, and other cognitive processes in order to form coherent memory representations.

### 4.3 Limitations

The spatial accuracy of scalp-level EEG activity and its source localization results are still a matter of debate. A large portion of memory-related neural activity happens in the subcortical regions, especially within the hippocampus (e.g. Geng et al., 2019; Ghetti et al., 2010; Ofen et al., 2007). However, there is a general agreement that scalp EEG has very limited access to subcortical activity, though some study has provided evidence that high-density scalp EEG can sense subcortical activities (Seeber et al., 2019). Considering the absence of robust validation methods for localized EEG sources (Michel and He, 2019), our analysis excluded all subcortical regions from the source analysis. Additionally, caution should be taken when interpreting source localization results in MTL regions given the potential range of error (Seeber et al., 2019) in localizing sources to the deeper regions of the brain that are far away from the scalp, where EEG signals were collected. While a lack of “confidence” measure restricts our ability to assess the reliability of the localized neural activity associated with any particular ERP component, it is necessary to interpret localization results alongside reasonable, a priori identified cortical ROIs.

Another limitation is associated with the nature of the LSW component. Unlike the P2 component, LSW exhibits a plateau-like slow increase after reaching local maximum, pointing to underlying oscillatory activities that tend to be anatomically widespread and reflect neural activity in a broadly distributed network. The lack of a “peak” in the LSW component makes it challenging to identify the appropriate time window to conduct source localization, especially across participants at different ages who may exhibit different latency of LSW (Geng et al., 2018, Rollins and Riggins, 2013). Adding on to the challenge is individual differences in sustained attention (Decker et al., 2023) and memory strategies (Sprondel et al., 2011) during late-stage memory encoding. Given these challenges, it is possible that LSW is contributed by other cortical regions in addition to MTL, and those results are not meeting significance threshold due to high signal-to-noise ratio.

### 4.4 Future directions

Future research may benefit from multimodal analysis in the time-frequency domain as well as with longitudinal approaches. Recent research is beginning to support source localization of EEG activity in specific frequency bands through estimated functional connectivity. Source localization of functional EEG connectivity represents a relatively novel direction (Xie et al., 2022), which has only recently become feasible due to advancements in computational power and modeling methods. This approach might also offer new insights into the underlying neural origins of slow wave activities such as LSW that are generally harder to localize.

Researchers should also consider further integrating EEG and fMRI modalities across different developmental stages, to explore the intricate relations among evolving neural dynamics. Historically, underdevelopment of MTL is considered to be a rate-limiting factor of episodic memory through MTL’s unique capacity to process contextual information (and other episodic features) associated with memory (e.g., Ghetti et al., 2010; see Ghetti and Bunge, 2012 for review). Our P2 source localization results hint that selective attention, a function related to the frontoparietal network and possibly the MTL as well, is one rate-limiting factor in the development of episodic memory encoding. Thus, the underdevelopment of MTL could “double-limit” memory capacity through both the attention mechanism and the memory binding mechanism. Unfortunately, we cannot test the developmental trajectory of the attentional process due to the limitation of this particular dataset. To further elucidate these dynamics, future studies should look at a broader age range and include longitudinal data to capture the evolution of attentional neural signals, and monitor their sources over time, to validate this idea. Overall, examining the temporal and developmental patterns of P2 and LSW components will help us better understand the developing mechanisms of episodic memory encoding. Longitudinal multimodal analysis is needed to shed a light on dynamic age-related changes among P2/LSW activities, their neural sources, and the matching fMRI activations during memory development.

## 5 Conclusion

The present study examined overlapping neural sources between EEG- and fMRI- identified cortical areas associated with source memory encoding. Both P2 and LSW were sensitive to source memory encoding. The timing differences in these components reveal information about encoding processes. The effects at the earlier P2 and later LSW suggest processes related to evaluating or integrating the source of memory are distributed across a wide timeframe. Their respective timing is consistent with previous work on the role of attention and its early impact on memory encoding, and the role of LSW in updating and integrating memory with details. We used EEG source localization to bridge between EEG and fMRI modalities, linking the timing of the two components to their cortical sources. Specifically, activations in the frontoparietal regions (Geng et al., 2021; reviewed by Spaniol et al., 2009) have been hypothesized to modulate memory encoding via attentional/cognitive control processes, but the timing of such interaction is difficult to assess through fMRI alone. Our analyses identified the sources of P2 effects in the frontoparietal regions, adding strong evidence that interaction between attention and memory occurs early on in the encoding process. The localization of LSW sources to MTL adds to our current understanding of MTL’s role in late-stage memory processing. Furthermore, the localization of P2 sources to MTL (in addition to being sources of LSW) encourages researchers to think more about the role MTL plays *early* in the encoding process, especially its interaction with frontoparietal control processes. Our multimodal approach cross-validated results from ERP and fMRI modalities and creates a “chain of evidence” for the neural origin, timing, and effect of the underlying neural signals.

Early childhood is a period requiring precision in both anatomical and temporal dimensions when identifying the changing neural signals associated with evolving memory (e.g., Geng et al., 2021). Comparing and combining findings from ERP and fMRI methods may shed new light on current literature of memory encoding, as it will help provide new constraints to the functionality and mechanisms behind the understudied ERPs of memory encoding. We hope that this report begins to bring the literatures on EEG and fMRI during memory development together and allow for a more comprehensive characterization of memory-related neural signatures.

## CRediT authorship contribution statement

**Yuqing Lei:** Writing – review & editing, Writing – original draft, Visualization, Validation, Methodology, Investigation, Formal analysis, Conceptualization. **John Richards:** Software, Resources, Methodology. **Fengji Geng:** Software, Methodology. **Tracy Riggins:** Writing – review & editing, Supervision, Resources, Project administration, Funding acquisition, Conceptualization.

## Declaration of Competing Interest

The authors declare that they have no known competing financial interests or personal relationships that could have appeared to influence the work reported in this paper.

## Data Availability

No new data was created.

## Appendix A Behavioral Analysis

Source memory performances from EEG and fMRI sessions were summarized using R and compared to previous reports using this dataset (Geng et al., 2018, Geng et al., 2019). Behavioral analysis of task performance across both EEG and fMRI recording sessions, as reported by prior publications (Geng et al., 2018, Geng et al., 2019), indicated a mean source memory accuracy of 0.53 (*SD* = 0.13) in the MRI session, and a mean source memory accuracy of 0.49 (*SD* = 0.11) in the ERP session. In this report, among children who performed both recording sessions, average number of days between the two sessions were 12.3 (*SD* = 12.63, *M*dn  = 7). Mean performances of this subset of children were consistent with previous reports of this dataset (Geng et al., 2018, Geng et al., 2019), with a mean source memory accuracy of 0.58 (*SD* = 0.15) in the MRI session, and a mean source memory accuracy of 0.49 (*SD* = 0.13) in the ERP session.

We additionally compared source memory performance between the first session and the second session to examine possible repetition effect on task performance. Paired *t*-test showed a significant decrease in *source memory accuracy* (i.e., correct source memory retrieval out of correctly recognized items), from the first session to the second session, *t*(40) = -2.44, *p* = 0.010. The decrease was driven by an increase in the number of correctly recognized items (i.e., correctly recognizing whether an item was “old” or “new”), *t*(40) = 3.04, *p* = 0.004. There were no significant between-session differences in *total source memory percentage* (i.e., percentage of correct source memory retrieval out of all trials).

To investigate whether children had consistent source memory performance between the two sessions, in spite of an improvement in recognition memory, we conducted a multiple regression using second-session total source memory percentage as the dependent variable, and first-session total source memory percentage as the predictor, controlling for age. First-session total source memory percentage strongly predicted second-session total source memory percentage, *β*(38) = 0.62, *p* < 0.001. This result suggests that differences in source memory performance were relatively stable between two recording sessions, despite the improvement in recognition memory.

## Appendix B Age-related Analysis

In the ERP analysis, we additionally examined age-related effects on P2 and LSW amplitudes. Mixed-effect linear models were used to model P2 and LSW amplitudes as a function of age, controlling for Condition, Coronal Plane, Sagittal Plane, and their interactions, with a random intercept for subjects to account for within-subject variability. No main effects of age were identified in either P2 or LSW. We then modeled P2 and LSW amplitudes as a function of the interaction among Age, Condition, Coronal Plane, and Sagittal Plane, with a random intercept for subjects. No interaction with age was identified in P2 amplitudes. There were marginally significant interactions between Age X Sagittal Plane (*t*(1674) = 1.785, p = 0.075), as well as Age X Sagittal Plane X Condition (*t*(1674) = -1.646, p = 0.100) in LSW. To further explore these interactions, we used a median split and categorized age into two age groups (following approach in Geng et al., 2018). Post hoc pairwise comparison examined all Age Group X Sagittal Plane pairs for conditional differences in LSW amplitudes, corrected through the Tukey method. In the younger group, LSW amplitude showed significant conditional differences in left (*t*(1581) = 3.754, *p* < 0.001) and middle (*t*(1581) = -3.240, *p* = 0.001) channel clusters. In the older group, LSW amplitude showed significant conditional differences in right channel clusters (*t*(1581) = -2.756, *p* = 0.006). These LSW results are consistent with previously reported age effects on LSW in Geng et al. (2018).

In the source analysis, we also examined age-related effects for P2 and LSW CDR source scores separately. A mixed-effect linear model was applied to each source ROI, modeling the CDR score of the ROI as a function of age, controlling for source memory condition. An alternative model was also tested, including an interaction term between Age and Condition. Both models included a random intercept for subjects. Multiple comparison corrections were conducted using BH procedure to correct for FDR. No age-related effects were identified for both P2 and LSW after correction in either model.
